# Glycine zinc sulfate penta­hydrate: redetermination at 10 K from time-of-flight neutron Laue diffraction

**DOI:** 10.1107/S2056989016014304

**Published:** 2016-09-16

**Authors:** A. Dominic Fortes, Christopher M. Howard, Ian G. Wood, Matthias J. Gutmann

**Affiliations:** a ISIS Facility, Rutherford Appleton Laboratory, Harwell Science and Innovation, Campus, Didcot, Oxfordshire OX11 0QX, England; b Department of Earth Sciences, University College London, Gower Street, London, WC1E 6BT, England

**Keywords:** crystal structure, glycine, zinc sulfate, zwitterion, dimer, neutron diffraction

## Abstract

Single crystals of glycine zinc sulfate penta­hydrate [systematic name: hexa­aqua­zinc tetra­aquadiglycinezinc bis­(sulfate)], [Zn(H_2_O)_6_][Zn(C_2_H_5_NO_2_)_2_(H_2_O)_4_](SO_4_)_2_, have been grown by isothermal evaporation from aqueous solution at room temperature and characterized by single-crystal neutron diffraction. The unit cell contains two unique ZnO_6_ octa­hedra on sites of symmetry -1 and two SO_4_ tetra­hedra with site symmetry 1; the octa­hedra comprise one [tetra­aqua-diglycine zinc]^2+^ ion (centred on one Zn atom) and one [hexa­aqua­zinc]^2+^ ion (centred on the other Zn atom); the glycine zwitterion, NH_3_^+^CH_2_COO^−^, adopts a monodentate coordination to the first Zn atom. All other atoms sit on general positions of site symmetry 1. Glycine forms centrosymmetric closed cyclic dimers due to N—H⋯O hydrogen bonds between the amine and carboxyl­ate groups of adjacent zwitterions and exhibits torsion angles varying from ideal planarity by no more than 1.2°, the smallest values for any known glycine zwitterion not otherwise constrained by a mirror plane. This work confirms the H-atom locations estimated in three earlier single-crystal X-ray diffraction studies with the addition of independently refined fractional coordinates and *U_ij_* parameters, which provide accurate inter­nuclear *X*—H (*X* = N, O) bond lengths and consequently a more accurate and precise depiction of the hydrogen-bond framework.

## Abstract 2

We report a redetermination based on single-crystal neutron diffraction data and Raman spectra for glycine zinc sulfate penta­hydrate.

## Chemical context   

Numerous coordination compounds of glycine (Glyc) with divalent metal sulfates are known. For the case of zinc, there is an anhydrous species, 2Glyc·ZnSO_4_ (Moldobaev & Nogoev, 1970 ▸) and two hydrates, Glyc·ZnSO_4_·3H_2_O and Glyc·ZnSO_4_·5H_2_O. The trihydrate is dimorphic, occurring either as an ortho­rhom­bic crystal (space group *Pca*2_1_) or as a monoclinic crystal (*P*2_1_/*n*) depending on the synthesis route (Fleck & Bohatý, 2004 ▸). The monoclinic form is isotypic with compounds of general formula Glyc·*M*(II)SO_4_·3H_2_O where *M*(II) = Mg, Co or Fe (Oguey *et al.*, 2013**a* ▸,b* ▸, 2014 ▸). Compounds with the general formula Glyc·*M*(II)SO_4_·5H_2_O are known only as isotypic triclinic crystals (*P* 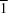) for *M*(II) = Mg, Mn, Co, Fe and Zn (Lindqvist & Rosenstein, 1960 ▸; Elayaraja *et al.*, 2007 ▸; Fleck & Bohatý, 2006 ▸; Tepavitcharova *et al.*, 2012 ▸). Solubility data have been published for a purported Glyc·NiSO_4_·5H_2_O (Moldobaev *et al.*, 1970 ▸; Alymkulova & Salyeva, 1987 ▸). We have collected as-yet unpublished X-ray powder-diffraction data from this species, showing that it is isotypic with the other known members of the series. The existence of Glyc·CuSO_4_·5H_2_O has been reported by Thilagavathi *et al.* (2012 ▸) but their work is in error, and quite unambiguously describes the well-known material CuSO_4_·5H_2_O.
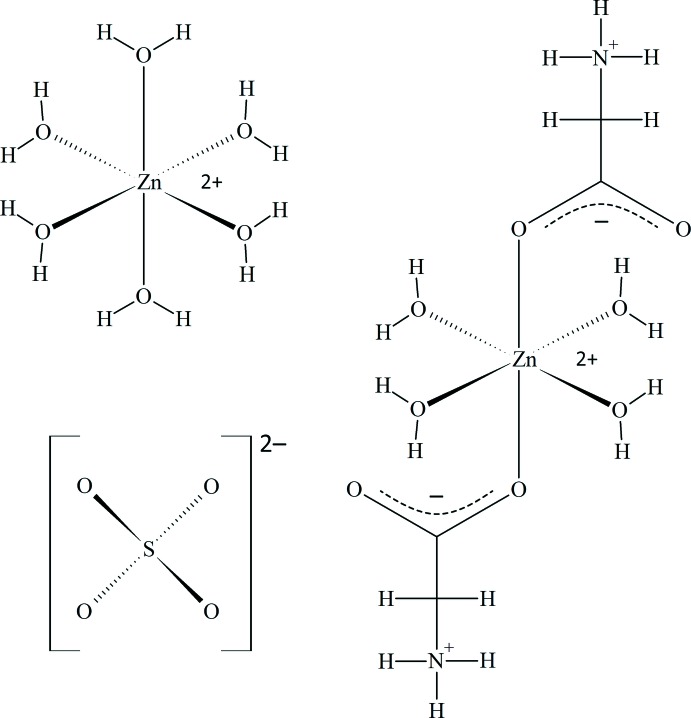


**Figure 1:**
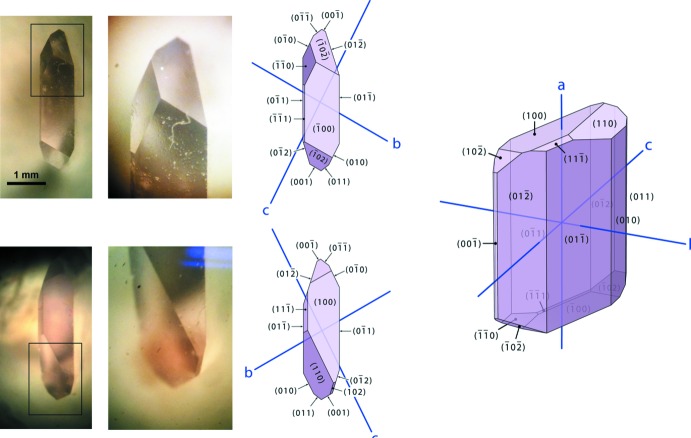
Microphotograph of a representative Glyc·ZnSO_4_·5H_2_O single crystal viewed along the *a* axis; insets show details of less well-developed facets (*e.g.*, 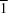 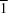
1 and 102). Drawings with each face labelled by the Miller index are shown on the right and a qu­anti­tative representation of the model is included in the CIF data. Figure produced and CIF code exported using *WinXMorph* (Kaminsky, 2005 ▸, 2007 ▸).

We recently carried out the first neutron diffraction study of Glyc·MgSO_4_·3H_2_O and Glyc·MgSO_4_·5H_2_O using perdeuterated powder specimens (Howard *et al.*, 2016 ▸). Glyc·MgSO_4_·5H_2_O tends to form masses of crystals that are both of poor quality and are too small for single-crystal neutron diffraction study; however, this is not the case for Glyc·MnSO_4_·5H_2_O and Glyc·ZnSO_4_·5H_2_O, where fine tabular to blocky single crystals with volumes substanti­ally in excess of 10 mm^3^ are formed with ease (Fig. 1 ▸). The objective of this work was to carry out the first single-crystal *neutron* diffraction study of any Glyc·*M*(II)SO_4_·5H_2_O compound, specifically using a specimen with *M*(II) = Zn.

Optical and mechanical properties of the title compound were reported by Balakrishnan & Ramamurthy (2007 ▸), although they incorrectly give the composition as Glyc·ZnSO_4_·7H_2_O. The effect of doping Glyc·ZnSO_4_·5H_2_O with cobalt is described by El-Fadl & Abdulwahab (2010 ▸). Three prior structure refinements from single-crystal X-ray diffraction data have been reported (Balamurugan *et al.*, 2011 ▸; Tepavitcharova *et al.*, 2012 ▸; Oguey *et al.*, 2013*c* ▸); comparisons with this work are detailed in Section 2.

## Structural commentary   

Although the stoichiometry of the material is accurately reflected in its common name, glycine zinc sulfate penta­hydrate, the presence of two symmetry-inequivalent Zn sites means that the crystallographically proper structural compos­ition is the ‘double’ formula [Gly·ZnSO_4_·5H_2_O]_2_, or more precisely [Zn(H_2_O)_6_][Zn(H_2_O)_4_(C_2_H_5_NO_2_)_2_](SO_4_)_2_; the unit cell contains *one* of these units.

**Figure 2:**
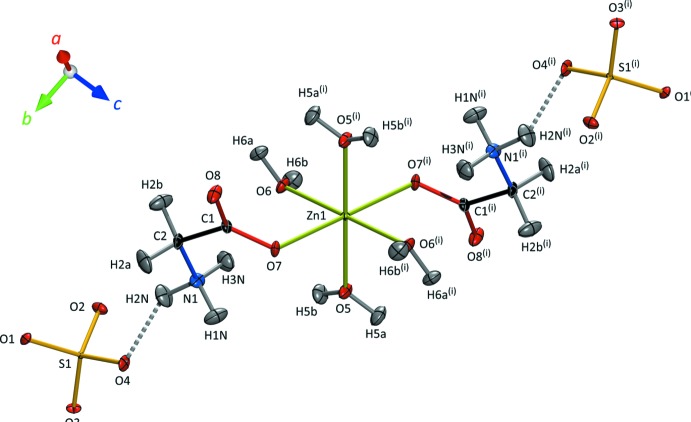
Local coordination environment of the Zn1 atom. Displacement ellipsoids are drawn at the 50% probability level for H and 90% for all other atoms. Dashed lines indicate N—H⋯O hydrogen bonds. [Symmetry code: (i) 1 − *x*, −*y*, 1 − *z*.]

**Figure 3:**
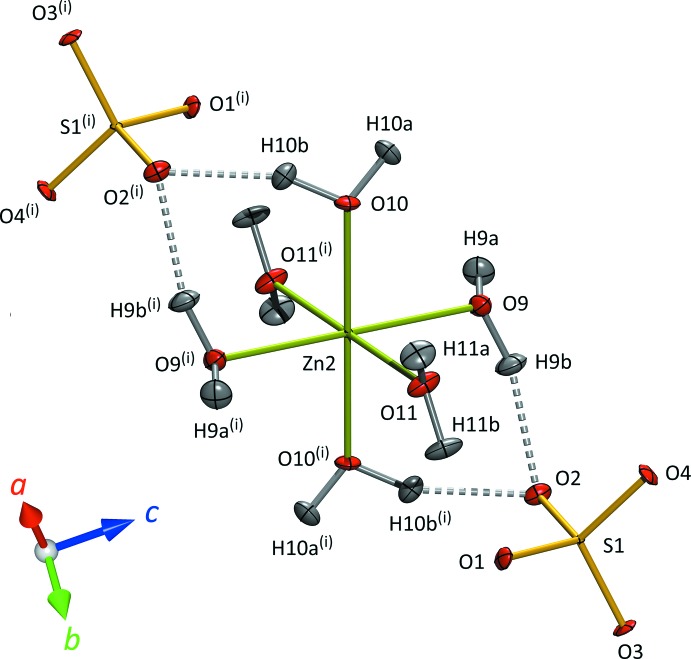
Local coordination environment of the Zn2 atom. Displacement ellipsoids are drawn at the 50% probability level for H and 90% for all other atoms. Dashed lines indicate O—H⋯O hydrogen bonds. [Symmetry code: (i) 1 − *x*, −*y*, 1 − *z*.]

The Zn1 coordination octa­hedron consists of tetra­aqua-diglycine zinc(II) with the glycine zwitterion (NH_3_^+^CH_2_COO^−^) coordinating to Zn by one of the carboxyl­ate oxygen atoms (Fig. 2 ▸); the inversion centre results in an all-*trans* configuration for these units. The Zn2 octa­hedron has the form hexa­aqua­zinc(II); the sulfate tetra­hedra are isolated, accepting hydrogen bonds primarily (but not exclusively) from Zn-coordinating water mol­ecules (Fig. 3 ▸). The S—O bond lengths (Table 1 ▸) reflect the number of hydrogen bonds accepted by each apical oxygen atom with a statistical significance which was not apparent from the powder refinement of Howard *et al.* (2016 ▸) but which are in excellent agreement with the single-crystal X-ray study of Tepavitcharova *et al.* (2012 ▸).

Bond lengths and angles of the glycine zwitterion agree very well with other determinations of related compounds made by X-ray single-crystal diffraction at higher temperatures and extremely well with the determinations in α-glycine at room temperature by neutron single-crystal diffraction (Jönsson & Kvick, 1972 ▸; Power *et al.*, 1976 ▸), particularly in respect of their mean N—H bond lengths (1.039 Å) and mean C—H bond lengths (1.090 Å). The glycine zwitterion is remarkably planar, with torsion angles O7—C1—C2—N1 = −1.18 (3)° and O8—C1—C2—N1 = 179.23 (2)°, even by comparison with, for example, glycine nickel bromide tetra­hydrate (Fleck & Bohatý, 2005 ▸), glycine lithium chromate monohydrate and glycine lithium molybdate (Fleck *et al.*, 2006 ▸), where torsion angles are in the range 169–176°. Only in glycine magnesium chloride tetra­hydrate, where the glycine zwitterion lies on a mirror plane, are values of 180° realized (Fleck & Bohatý, 2005 ▸). In α-glycine, the equivalent torsion angles are −19.60 (3) and 161.28 (2)°.

In respect of the heavy atoms, agreement in the inter­atomic distances and angles between the single-crystal X-ray and single-crystal neutron refinements (Table 1 ▸) is excellent, with some differences emerging in respect of the room-temperature refinement by Balamurugan *et al.* (2011 ▸). However, the neutron data provide a substantial improvement in accuracy with respect to the X-ray data in the hydrogen atom’s fractional coordinates and *U_ij_* parameters. This is not surprising since neutrons are sensitive to the nuclear positions and X-rays to the electron density; in the covalent *X*—H bond the centroid of the H-atom’s electron-density distribution is displaced towards the heavy atom by 0.1 Å, yielding *X*—H distances from 10–15% shorter than the true inter­nuclear separation (Coppens, 1997 ▸). Table 2 ▸ compares *X*—H bond lengths from a range of Gly·*M*(II)SO_4_·5H_2_O crystals obtained by single-crystal X-ray diffraction and by neutron powder diffraction. In the work of Elayaraja *et al.* (2007 ▸), methyl hydrogens were positioned geometrically and allowed to ride with *U*_iso_(H) = 1.2*U*_eq_(C); water hydrogen atoms were refined with restraints; the N—H bond lengths were all restrained to be equal. Balamurugan *et al.* (2011 ▸) placed the majority of their hydrogen atoms geometrically, although failed to identify the third amine hydrogen atom; water and the two amine H atoms were refined isotropically whereas the two methyl hydrogen atoms were riding on the C atom. Tepavitcharova *et al.* (2012 ▸) placed the hydrogen atoms in Gly·ZnSO_4_·5H_2_O geometrically and treated all of them as riding on their associated heavy atom during refinement. Oguey *et al.* (2013*c* ▸) allowed all water hydrogen atoms to refine isotropically but fixed the methyl and amine hydrogens to ride on C and N, respectively. The coordinates of hydrogen atoms in this work were unrestrained and allowed to refine anisotropically.

Our values for the N—H and C—H bond lengths are in excellent agreement with other single-crystal neutron diffraction work, as noted in the preceding paragraph. Our values for the O—H bond lengths also agree well with those found in similar environments in hydrated *M*(II) coordination compounds, such as MgSO_4_·11H_2_O and MgCrO_4_·11H_2_O where the average O–H = 0.974 Å (Fortes *et al.*, 2013 ▸), MgSeO_4_·9H_2_O, O—H_av_ = 0.972 Å (Fortes *et al.*, 2015 ▸), and MgSeO_4_·7H_2_O, O—H_av_ = 0.974 Å (Fortes & Gutmann, 2014 ▸).

## Supra­molecular features   

**Figure 4:**
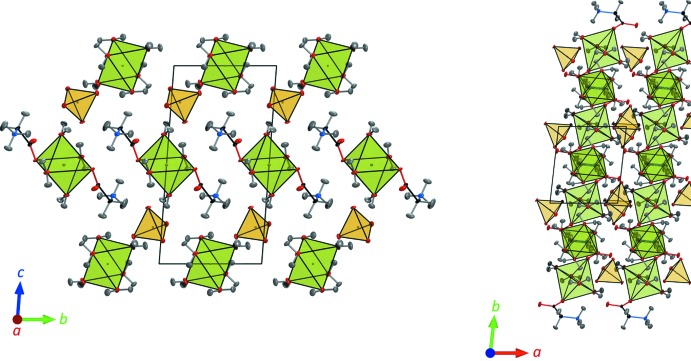
Packing of polyhedra in the structure of Glyc·ZnSO_4_·5H_2_O viewed along *a* (left) and along *c* (right). ZnO_6_ octa­hedra are green, SO_4_ tetra­hedra are yellow.

The overall three-dimensional framework is completed by a variety of hydrogen bonds with a range of strengths (Table 3 ▸). Fig. 4 ▸ shows the spatial relationship of the main structural elements. The majority of the hydrogen bonds are O—H⋯O contacts of medium strength (1.66 < H⋯O < 1.90 Å) and high linearity (∠ O—H⋯O > 157°), characteristic of two-centred inter­actions. As expected, the N—H⋯O hydrogen bonds are weaker (*i.e*., longer, 1.85 < H⋯O < 2.22 Å) and more strained (∠ N—H⋯O between approx. 140–160°). The methyl groups appear to participate in weak C—H⋯O hydrogen bonds (*cf*., Steiner & Desiraju, 1998 ▸). One C—H⋯O bond is evidently a two-centred inter­action, being the shortest and most of linear contact of this kind, with H⋯O = 2.58 (1) Å and ∠ C—H⋯O = 167 (1)°. The other, involving C—H2*A*, is clearly a two-centred inter­action (*i.e*., a bifurcated hydrogen bond) with ‘arms’ of roughly equal length, H⋯O ≃ 2.7 Å and C—H⋯O angles of 119 and 128° involving O2 and O5, respectively.

### Glycine dimers   

**Figure 5:**
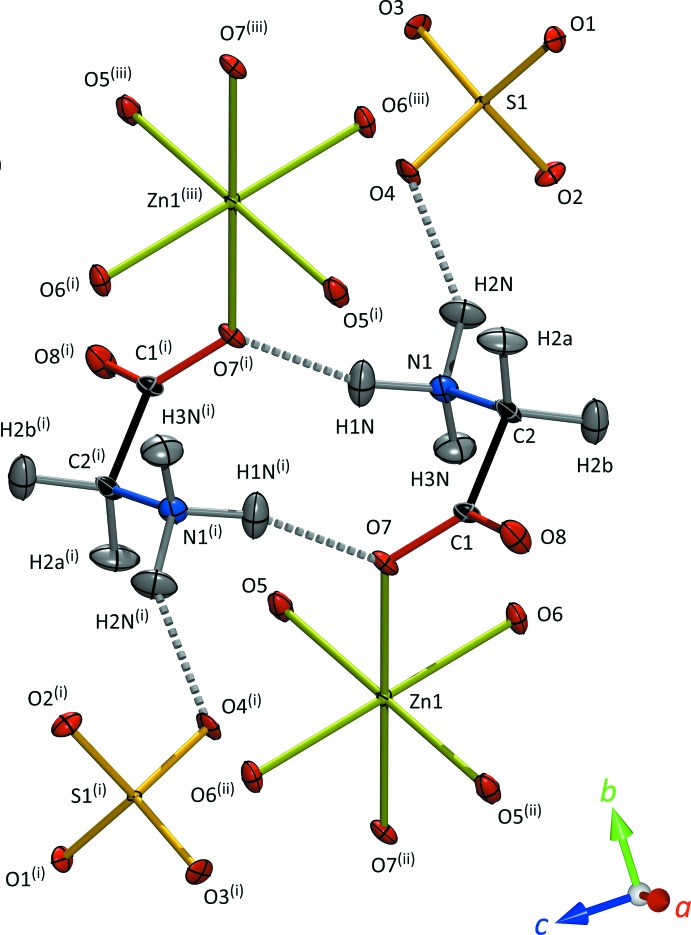
Connectivity between adjacent Zn1 octa­hedra is *via* a closed cyclic glycine dimer. As before, displacement ellipsoids are drawn at the 50% probability level for H and 90% for all other atoms. Dashed lines indicate N—H⋯O hydrogen bonds. [Symmetry codes: (i) 1 − *x*, 1 − *y*, 1 − *z*; (ii) 1 − *x*, −*y*, 1 − *z*; (iii) *x*, 1 + *y*, *z*.]

**Figure 6:**
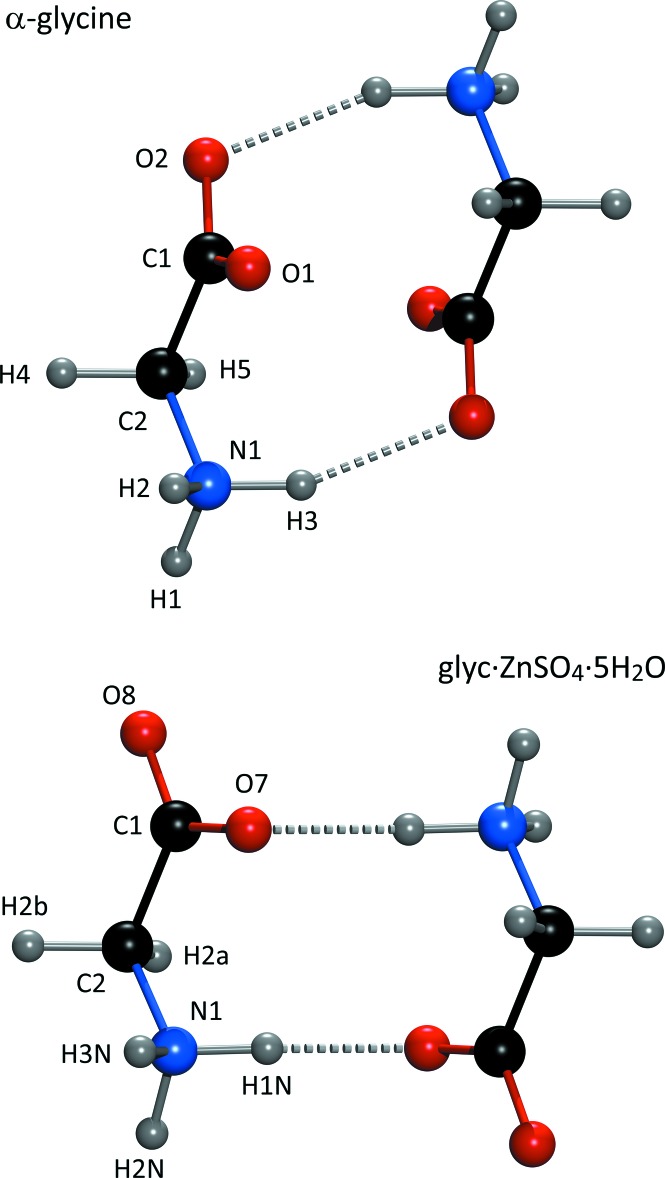
Comparison of the closed cyclic dimers involving zwitterionic glycine that occur in the crystal structures of α-glycine (top) and in Glyc·ZnSO_4_·5H_2_O (bottom).

A hitherto unrecognized aspect of the supra­molecular structure of Glyc·*M*(II)SO_4_·5H_2_O compounds is the presence of glycine dimers (Fig. 5 ▸). These occur as closed cyclic structures formed by N—H⋯O hydrogen bonds between the amine group of one glycine zwitterion and the Zn-coordin­ating carboxyl­ate oxygen (O7) of another zwitterion, related to the first by an inversion centre. A similar cyclic dimer occurs in the structure of α-glycine. A direct comparison between the dimers in Glyc·ZnSO_4_·5H_2_O and in α-glycine is shown in Fig. 6 ▸; clearly, the main difference between these two dimers is the orientation of the carboxyl­ate group, which is presumably due to the influence of a divalent metal being coordinated by the O7 carboxyl­ate oxygen. Experimental studies of aqueous solutions indicate that only glycine monomers exist in the liquid phase (Huang *et al.*, 2008 ▸). However, there has been widespread disagreement on this matter from computational studies, which indicate either that there are no dimers (Hamad & Catlow, 2011 ▸), substantial qu­anti­ties of closed zwitterionic dimers (Friant-Michel & Ruiz-López, 2010 ▸), or a small fraction of open dimers (Yani *et al.*, 2012 ▸) present in saturated solutions. The presence or absence of glycine polymerization in coordination compounds such as these may be useful in understanding the association of glycine in saturated aqueous solutions during nucleation and the role of solvated metal ions in polymerizing amino acids in Earth’s Hadean oceans (Kitadai *et al.*, 2011 ▸, 2016 ▸) or in extraterrestrial oceans elsewhere in our solar system (Kimura & Kitadai, 2015 ▸).

## Database survey   

A search of the Cambridge Structural Database (Groom *et al.*, 2016 ▸) identified the following directly relevant entries:

Glyc·*M*(II)SO_4_ penta­hydrates: 672589 (Mg); 857075 (Mg); 1451396 (Mg); 296329 (Co); 857073 (Co); 806684 (Zn); 857076 (Zn); 936400 (Zn).

Glyc·*M*(II)SO_4_ trihydrates: 989590 (Mg); 1451397 (Mg); 857074 (Co); 936396 (Fe); 243588 (Zn, ortho­rhom­bic); 936394 (Zn, monoclinic).

Glyc·*M*(II)SO_4_ hexa­hydrates: 1285639 (Ni).

## Vibrational spectroscopy   

Laser-stimulated Raman spectra were measured using a portable B&WTek *i*-Raman Plus spectrometer equipped with a 532 nm laser (*P*_max_ = 37 mW at the probe tip) that records spectra over the range 171–4002 cm^−1^ with an optimal resolution of 3 cm^−1^. Measurements were carried out on powdered specimens of α-glycine and Glyc·ZnSO_4_·5H_2_O. Samples were measured in thin-walled glass vials using the BC100 fibre-optic coupled Raman probe; the total integration time and laser power for each sample is provided with the tabulated results (see supplementary material).

**Figure 7:**
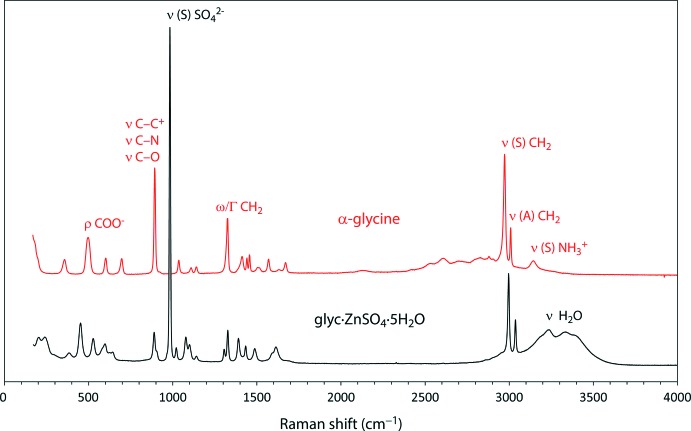
Raman spectra of α-glycine (top) and Glyc·ZnSO_4_·5H_2_O (bottom). Selected vibrational modes are labelled and a complete qu­anti­tative listing is given in Table 4 ▸.

The Raman spectrum of Glyc·ZnSO_4_·5H_2_O (Fig. 6 ▸) is virtually identical with that of Glyc·MgSO_4_·5H_2_O reported in Howard *et al.* (2016 ▸) and is in excellent agreement with the spectrum shown in Tepavitcharova *et al.* (2012 ▸). Numerical data of the Raman spectrum are provided as an electronic supplement; peak positions and vibrational mode assignments are given in Table 4 ▸. The main differences between the two divalent-metal-substituted compounds include the blue-shifting of octa­hedral deformation modes and blue-shifting of both symmetric and asymmetric COO^−^ stretching modes. A large blue-shift of ν(*A*) and ν(*S*) COO^−^ occurs when glycine coordinates to Mg^2+^ and the shift increases when glycine coordinates to Zn^2+^. Raman spectra of α-glycine and Glyc·ZnSO_4_·5H_2_O are shown in Fig. 7 ▸.

## Synthesis and crystallization   

Glyc·ZnSO_4_·5H_2_O was crystallized by evaporation at room temperature of an equimolar aqueous solution of α-glycine (Alfa Aesar A13816) and ZnSO_4_·7H_2_O (Sigma Aldrich Z4750) in deionized water (Alfa Aesar 36645). Unlike the MgSO_4_-bearing analogue, Glyc·ZnSO_4_·5H_2_O forms large well-faceted crystals that are both amenable to morphological study and suitably large for single-crystal neutron diffraction analysis. Fig. 1 ▸ shows photographs of a representative crystal viewed along its *a* axis and series of drawings with indexed crystal faces.

## Data collection and refinement   

Crystal data, data collection and structure refinement details are summarized in Table 5 ▸. Data were collected from a pair of single crystals at a series of four discrete rotational positions about the vertical axis, each frame being counted for 5 h, equivalent to 800 µAhr of ISIS proton beam current per frame. The structure of Glyc(*d*_5_)·MgSO_4_·5D_2_O at 10 K reported by Howard *et al.* (2016 ▸) was used as a starting point for the refinement. A total of eleven peaks, with the largest σ(*F*_obs_–*F*_calc)_ values were omitted from the refinement; such outliers are fairly common in SXD measurement when peaks occur close to the edges of detectors. A mild restraint on the *U_ij_* parameters of the sulfur atom was imposed (*SHELX* ISOR command) in order to avoid a slightly non-positive-definite displacement ellipsoid. Since sulfur has the smallest neutron scattering cross section of any atom in the structure, and since it is both comparatively heavy and the temperature is very low, it is not surprising that – within errors – the effective *U*_iso_ parameter should refine to a small negative value.

## Supplementary Material

Crystal structure: contains datablock(s) I. DOI: 10.1107/S2056989016014304/wm5318sup1.cif

Structure factors: contains datablock(s) I. DOI: 10.1107/S2056989016014304/wm5318Isup2.hkl

Raman spectrum of glycine zinc sulfate pentahydrate. Raman shift is in units of reciprocal centimetre and the intensity is in arbitrary units.. DOI: 10.1107/S2056989016014304/wm5318sup3.txt

CCDC reference: 1503478

Additional supporting information: crystallographic information; 3D view; checkCIF report

## Figures and Tables

**Table 1:** Comparison of bond lengths (Å), polyhedral volumes (Å^3^) and various distortion metrics (*cf*., Robinson *et al.*, 1971 ▸) in Glyc·ZnSO_4_·5H_2_O from this work and the three preceding single-crystal X-ray diffraction studies The distortion index and quadratic elongation are dimensionless, whereas the bond-angle variance is in units of degrees squared.

	This work	Balamurugan *et al.* (2011 ▸)	Tepavitcharova *et al.* (2012 ▸)	Oguey *et al.* (2013*c* ▸)
	Single-crystal neutron	Single-crystal X-ray	Single-crystal X-ray	Single-crystal X-ray
	*T* = 10 K	*T* = 293 K	*T* = 150 K	*T* = 153 K
S—O1*	1.474 (5)	1.472 (2)	1.472 (1)	1.473 (2)
S—O2	1.484 (4)	1.478 (2)	1.482 (1)	1.485 (2)
S—O3*	1.473 (4)	1.472 (2)	1.477 (1)	1.481 (2)
S—O4	1.480 (5)	1.481 (2)	1.484 (1)	1.479 (2)
Mean S—O	1.478	1.476	1.479	1.479
SO_4_ volume	1.656	1.649	1.659	1.661
Distortion index	0.0028	0.0025	0.0027	0.0022
Quadratic elongation	1.000	1.000	1.000	1.000
Bond-angle variance	0.410	0.268	0.320	0.420
				
Zn1—O5	2.039 (2)	2.024 (3)	2.032 (1)	2.035 (2)
Zn1—O6	2.093 (2)	2.101 (3)	2.098 (1)	2.098 (2)
Zn1—O7^†^	2.173 (2)	2.181 (3)	2.177 (1)	2.176 (2)
Mean Zn1—O	2.102	2.102	2.102	2.103
ZnO_6_ volume	12.338	12.339	12.339	12.336
Distortion index	0.0227	0.0251	0.0238	0.0232
Quadratic elongation	1.003	1.003	1.003	1.003
Bond-angle variance	6.308	4.815	5.975	6.292
				
Zn2—O9	2.129 (3)	2.141 (3)	2.133 (1)	2.135 (2)
Zn2—O10	2.067 (3)	2.071 (3)	2.070 (1)	2.072 (2)
Zn2—O11	2.075 (2)	2.063 (3)	2.065 (2)	2.065 (2)
Mean Zn2—O	2.090	2.092	2.089	2.091
ZnO_6_ volume	12.127	12.176	12.123	12.145
Distortion index	0.0124	0.0156	0.0139	0.0142
Quadratic elongation	1.003	1.002	1.002	1.002
Bond-angle variance	7.982	5.942	6.617	6.541
				
C1—O7	1.272 (4)	1.272 (5)	1.274 (2)	1.278 (3)
C1—O8	1.240 (3)	1.228 (5)	1.236 (2)	1.234 (3)
C1—C2	1.523 (4)	1.516 (5)	1.525 (3)	1.522 (3)
C2—N1	1.481 (2)	1.478 (5)	1.480 (2)	1.480 (3)
				

*Denotes sulfate O atoms accepting two hydrogen bonds instead of three. ^†^Denotes carboxyl­ate oxygen ligand instead of water oxygen.

**Table 2:** Comparison of *X*—H(D) bond lengths (Å) from earlier work (*a*–*e*) with our own (*f*) Element symbols indicate the cation in each compound. ‘X-ray’ denotes single-crystal X-ray diffraction; ‘NPD’ denotes a neutron powder diffraction experiment on a deuterated analogue carried out at 10 K; ‘neutron’ indicates single-crystal neutron diffraction on a protonated analogue carried out at 10 K. Note that the atom symbols employed in our work are the same as those used by Elayaraja *et al.* (2007 ▸) and by Howard *et al.* (2016 ▸). Although other authors have used different atom labels – and indeed use them inconsistently in their own reports – we list equivalent contacts in this table.

	Mg, X-ray^(*a*)^	Mg, NPD^(*b*)^	Co, X-ray^(*c*)^	Mg, X-ray^(*c*)^	Zn, X-ray^(*c*)^	Zn, X-ray^(*d*)^	Zn, X-ray^(*e*)^	Zn, neutron^(*f*)^
N—H1N	0.87 (4)	1.008 (4)	0.847 (1)	0.849 (1)	0.881 (2)	0.85 (2)	0.910 (2)	1.033 (7)
N—H2N	0.87 (4)	0.982 (4)	0.907 (1)	0.898 (1)	0.904 (1)	0.83 (3)	0.911 (2)	1.028 (8)
N—H3N	0.87 (5)	0.991 (5)	0.904 (1)	0.902 (1)	0.946 (1)	absent	0.910 (2)	1.022 (6)
Average N—H	0.87	0.995	0.877	0.874	0.892	0.84	0.911	1.030
								
C—H2A	0.970 (4)	1.077 (4)	0.961 (1)	0.960 (1)	0.967 (2)	0.970 (3)	0.990 (2)	1.085 (6)
C—H2B	0.970 (3)	1.083 (4)	0.901 (1)	1.014 (1)	1.050 (2)	0.970 (3)	0.990 (2)	1.091 (7)
Average C—H	0.970	1.080	0.931	0.987	1.009	0.970	0.990	1.088
								
O5—H5A	0.84 (3)	0.975 (5)	0.880 (1)	0.789 (1)	0.879 (2)	0.85 (2)	0.83 (3)	0.973 (7)
O5—H5B	0.85 (3)	0.946 (5)	0.914 (1)	0.930 (1)	0.838 (1)	0.85 (3)	0.85 (3)	0.997 (7)
O6—H6A	0.84 (2)	0.987 (5)	0.964 (1)	0.875 (1)	0.864 (1)	0.83 (3)	0.86 (3)	0.981 (6)
O6—H6B	0.83 (3)	0.988 (5)	0.906 (1)	0.897 (1)	0.886 (1)	0.84 (3)	0.85 (2)	0.985 (6)
O9—H9A	0.83 (2)	0.977 (5)	0.864 (1)	0.871 (1)	0.881 (2)	0.87 (3)	0.86 (2)	0.979 (5)
O9—H9B	0.84 (2)	0.984 (4)	0.884 (1)	0.901 (1)	0.964 (1)	0.87 (2)	0.87 (3)	0.966 (6)
O10—H10A	0.84 (4)	0.954 (5)	0.972 (1)	0.911 (1)	0.887 (1)	0.82 (2)	0.87 (2)	0.977 (8)
O10—H10B	0.84 (3)	0.972 (5)	0.855 (1)	0.821 (1)	0.913 (1)	0.84 (2)	0.85 (2)	0.978 (6)
O11—H11A	0.84 (3)	1.002 (5)	0.822 (1)	0.884 (1)	0.808 (1)	0.83 (3)	0.86 (2)	0.966 (6)
O11—H11B	0.83 (3)	0.965 (5)	0.906 (1)	0.859 (1)	0.900 (1)	0.84 (2)	0.84 (2)	0.966 (6)
Average O—H	0.84	0.975	0.897	0.874	0.882	0.85	0.85	0.977

(*a*) Elayaraja *et al.* (2007 ▸); (*b*) Howard *et al.* (2016 ▸); (*c*) Tepavitcharova *et al.* (2012 ▸); (*d*) Balamurugan *et al.* (2011 ▸); (*e*) Oguey *et al.* (2013*c* ▸); (*f*) this work.

**Table 3:** Hydrogen-bond geometry (Å, °)

*D*—H⋯*A*	*D*—H	H⋯*A*	*D*⋯*A*	*D*—H⋯*A*
O5—H5*A*⋯O4^i^	0.973 (7)	1.793 (7)	2.755 (4)	169.0 (7)
O5—H5*B*⋯O8^ii^	0.997 (7)	1.656 (8)	2.642 (4)	168.9 (6)
O6—H6*A*⋯O3^iii^	0.981 (6)	1.722 (6)	2.696 (3)	170.8 (5)
O6—H6*B*⋯O4^iv^	0.985 (6)	1.751 (5)	2.729 (3)	171.8 (7)
O9—H9*A*⋯O1^iv^	0.979 (5)	1.732 (5)	2.707 (3)	173.8 (6)
O9—H9*B*⋯O2	0.966 (6)	1.895 (6)	2.811 (3)	157.2 (6)
O10—H10*A*⋯O3^iii^	0.977 (8)	1.740 (8)	2.713 (4)	173.0 (7)
O10—H10*B*⋯O2^v^	0.979 (6)	1.811 (7)	2.745 (4)	158.5 (7)
O11—H11*A*⋯O2^vi^	0.966 (6)	1.772 (6)	2.726 (3)	168.6 (6)
O11—H11*B*⋯O1	0.966 (6)	1.824 (6)	2.750 (3)	159.5 (7)
C2—H2*A*⋯O2^vi^	1.085 (6)	2.682 (9)	3.351 (4)	119.4 (6)
C2—H2*A*⋯O5^vii^	1.085 (6)	2.716 (8)	3.489 (3)	127.9 (6)
C2—H2*B*⋯O10	1.091 (7)	2.579 (8)	3.649 (4)	166.7 (7)
N1—H1*N*⋯O7^vii^	1.033 (7)	1.853 (7)	2.848 (3)	160.8 (7)
N1—H2*N*⋯O4	1.027 (8)	1.961 (7)	2.877 (3)	147.0 (7)
N1—H3*N*⋯O6	1.022 (6)	2.216 (7)	3.066 (3)	139.5 (5)

Symmetry codes: (i) 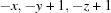; (ii) 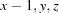; (iii) 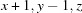; (iv) 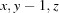; (v) 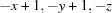; (vi) 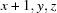; (vii) 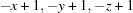.

**Table 4:** Raman vibrational frequencies and mode assignments of α-glycine (*cf*., Stenbäck, 1976 ▸: Rosado *et al.*, 1998 ▸: Yang *et al.*, 2008 ▸), Glyc·MgSO_4_·5H_2_O (Howard *et al.*, 2016 ▸) and the title compound Meaning of symbols: ν = stretch; δ = deformation; ρ = rock; ω = wag; Γ = twist; (*A*) = asymmetric; (*S*) = symmetric.

	α-Glycine^*a*^	Glyc·MgSO_4_·5H_2_O^*a*^	Glyc·ZnSO_4_·5H_2_O
Vibrational mode	180 s, 18 mW	1400 s, 18 mW	540 s, 18 mW
			
δ *M* ^2+^—O (?)	–	208	203
	–	236	220
			
δ CCN^+^	356	361	382
ρ COO^−^			
			
δ(*S*) SO_4_^2−^	–	453	451
ρ COO^−^	497	522	527
ω COO^−^	601	597	582
			599
			
δ(*A*) SO_4_^2−^		623	626
		645	644
			
δ COO^−^	696	–	–
unknown	–	794	–
			
ν C—C^+^	893	890	890
ν C—N		905	906
ν C—O			
			
ρ CH_2_	922	–	–
ν(*S*) SO_4_^2−^	–	983.8	983.2
ν C—N	1036	1020	1021
			
			
ν(*A*) SO_4_^2−^	–	1077	1078
		1100	1101
			
ρ NH^3+^	1108	1139	1141
	1140		
ω CH_2_	1325	1305	1306
Γ CH_2_		1328	1327
			
ν(*S*) COO^−^	1410	1395	1391
			
δ(*S*) CH_2_	1441	1434	1433
	1457		
			
δ(*A*) NH^3+^	1502		
δ(*S*) NH^3+^	1516	1488	1488
	1569		
			
ν C—C^+^	1634	1597	1590
ω CH_2_			
			
ν(*A*) COO^−^	1670	1631	1614
ν(*S*) CH_2_	2972	2997	2996
ν(*A*) CH_2_	3009	3038	3037
ν(*S*) NH^3+^	3143	–	–
			
ν(*S*) H_2_O	–	3248	3204
			3233
			
ν(*A*) H_2_O	–	3384	3331
			3405

^*a*^Howard *et al.* (2016 ▸).

**Table 5:** Experimental details

Crystal data	
Chemical formula	[Zn(H_2_O)_6_][Zn(C_2_H_5_NO_2_)_2_(H_2_O)_4_](SO_4_)_2_
*M*_r_	653.20
Crystal system, space group	Triclinic, *P* 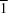
Temperature (K)	10
*a*, *b*, *c* (Å)	5.9601 (15), 6.7670 (17), 13.112 (4)
α, β, γ (°)	84.955 (18), 83.25 (2), 83.042 (19)
*V* (Å^3^)	519.8 (2)
*Z*	1
Radiation type	Neutron, λ = 0.48-7.0 Å
μ (mm^−1^)	5.02 + 0.0182 * λ
Crystal size (mm)	4 × 2.5 × 1

Data collection	
Diffractometer	SXD
Absorption correction	Numerical. The linear absorption coefficient is wavelength dependent and is calculated as: μ = 5.0165 + 0.0182 * λ [cm^-1^] as determined by Gaussian integration in *SXD2001* (Gutmann, 2005 ▸)
No. of measured, independent and observed [*I* > 2σ(*I*)] reflections	8296, 8296, 8296
*R*_int_	0.089

Refinement	
*R*[*F* ^2^ > 2σ(*F* ^2^)], *wR*(*F* ^2^), *S*	0.089, 0.246, 1.09
No. of reflections	8296
No. of parameters	291
No. of restraints	12
H-atom treatment	All H-atom parameters refined
Δρ_max_, Δρ_min_ (e Å^−3^)	3.20, −3.47

Computer programs: *SXD2001* (Gutmann, 2005 ▸), *SHELXT2014* (Sheldrick, 2015*a* ▸; Gruene *et al.*, 2014 ▸), *SHELXL2014* (Sheldrick, 2015*b* ▸; Gruene *et al.*, 2014 ▸), *DIAMOND* (Putz & Brandenburg, 2006 ▸) and *publCIF* (Westrip, 2010 ▸).

## supplementary crystallographic information

### Crystal data

**Table:**

[Zn(H_2_O)_6_][Zn(C_2_H_5_NO_2_)_2_(H_2_O)_4_](SO_4_)_2_	*Z* = 1
*M**_r_* = 653.20	*F*(000) = 336
Triclinic, *P*1	*D*_x_ = 2.086 Mg m^−^^3^
*a* = 5.9601 (15) Å	Neutron radiation, λ = 0.48-7.0 Å
*b* = 6.7670 (17) Å	Cell parameters from 550 reflections
*c* = 13.112 (4) Å	µ = 5.02 + 0.0182 * λ mm^−^^1^
α = 84.955 (18)°	*T* = 10 K
β = 83.25 (2)°	Tabular, colourless
γ = 83.042 (19)°	4 × 2.5 × 1 mm
*V* = 519.8 (2) Å^3^	

### Data collection

**Table:**

SXD diffractometer	8296 reflections with *I* > 2σ(*I*)
Radiation source: ISIS neutron spallation source	*R*_int_ = 0.089
time–of–flight LAUE diffraction scans	θ_max_ = 87.4°, θ_min_ = 8.2°
Absorption correction: numerical The linear absorption coefficient is wavelength dependent and is calculated as: µ = 5.0165 + 0.0182 * λ [cm^-1^] as determined by Gaussian integration in *SXD2001* (Gutmann, 2005)	*h* = −15→15
	*k* = −18→16
8296 measured reflections	*l* = −28→29
8296 independent reflections	

### Refinement

**Table:**

Refinement on *F*^2^	Hydrogen site location: difference Fourier map
Least-squares matrix: full	All H-atom parameters refined
*R*[*F*^2^ > 2σ(*F*^2^)] = 0.089	*w* = 1/[σ^2^(*F*_o_^2^) + (0.1376*P*)^2^ + 36.2519*P*] where *P* = (*F*_o_^2^ + 2*F*_c_^2^)/3
*wR*(*F*^2^) = 0.246	(Δ/σ)_max_ < 0.001
*S* = 1.09	Δρ_max_ = 3.20 e Å^−^^3^
8296 reflections	Δρ_min_ = −3.47 e Å^−^^3^
291 parameters	Extinction correction: SHELXL, Fc^*^=kFc[1+0.001xFc^2^λ^3^/sin(2θ)]^-1/4^
12 restraints	Extinction coefficient: 0.0386 (18)

### Special details

**Table:**

Experimental. For peak integration a local UB matrix refined for each frame, using approximately 50 reflections from each of the 11 detectors. Hence _cell_measurement_reflns_used 550 For final cell dimensions a weighted average of all local cells was calculated Because of the nature of the experiment, it is not possible to give values of theta_min and theta_max for the cell determination. The same applies for the wavelength used for the experiment. The range of wavelengths used was 0.48–7.0 Angstroms, BUT the bulk of the diffraction information is obtained from wavelengths in the range 0.7–2.5 Angstroms. The data collection procedures on the SXD instrument used for the single-crystal neutron data collection are most recently summarized in the Appendix to the following paper Wilson, C.C. (1997). J. Mol. Struct. 405, 207–217
Geometry. All esds (except the esd in the dihedral angle between two l.s. planes) are estimated using the full covariance matrix. The cell esds are taken into account individually in the estimation of esds in distances, angles and torsion angles; correlations between esds in cell parameters are only used when they are defined by crystal symmetry. An approximate (isotropic) treatment of cell esds is used for estimating esds involving l.s. planes.
Refinement. The variable wavelength nature of the data collection procedure means that sensible values of _diffrn_reflns_theta_min & _diffrn_reflns_theta_max cannot be given instead the following limits are given _diffrn_reflns_sin(theta)/lambda_min 0.06 _diffrn_reflns_sin(theta)/lambda_max 1.38 _refine_diff_density_max/min is given in Fermi per angstrom cubed not electons per angstrom cubed. Another way to consider the _refine_diff_density_ is as a percentage of the scattering density of a given atom: _refine_diff_density_max = 5.7 % of hydrogen _refine_diff_density_min = -6.1 % of hydrogen Refinement of *F*^2^ against ALL reflections. The weighted *R*-factor *wR* and goodness of fit *S* are based on *F*^2^, conventional *R*-factors *R* are based on *F*, with *F* set to zero for negative *F*^2^. The threshold expression of *F*^2^ > σ(*F*^2^) is used only for calculating *R*-factors(gt) *etc*. and is not relevant to the choice of reflections for refinement. *R*-factors based on *F*^2^ are statistically about twice as large as those based on *F*, and *R*- factors based on ALL data will be even larger.

### Fractional atomic coordinates and isotropic or equivalent isotropic displacement parameters (Å^2^)

**Table:**

	*x*	*y*	*z*	*U*_iso_*/*U*_eq_	
S1	0.0141 (6)	0.9128 (6)	0.1847 (4)	0.0008 (6)	
O1	0.1809 (4)	1.0196 (3)	0.1159 (2)	0.0036 (4)	
O2	−0.0249 (4)	0.7303 (3)	0.1375 (2)	0.0037 (4)	
O3	−0.2027 (4)	1.0416 (3)	0.2010 (2)	0.0040 (4)	
O4	0.1050 (4)	0.8522 (4)	0.2845 (2)	0.0038 (3)	
Zn1	0.5000	0.0000	0.5000	0.0017 (4)	
O5	0.1954 (4)	0.1618 (4)	0.5392 (2)	0.0049 (4)	
H5A	0.0913 (11)	0.1389 (11)	0.6009 (6)	0.0198 (11)	
H5B	0.0998 (10)	0.2109 (9)	0.4826 (6)	0.0170 (10)	
O6	0.4360 (4)	0.0464 (4)	0.3456 (2)	0.0043 (4)	
H6A	0.5654 (10)	0.0305 (9)	0.2922 (5)	0.0162 (10)	
H6B	0.3082 (10)	−0.0116 (10)	0.3229 (6)	0.0179 (11)	
O7	0.6512 (4)	0.2793 (4)	0.4768 (2)	0.0037 (3)	
O8	0.9812 (4)	0.3195 (4)	0.3809 (2)	0.0060 (4)	
N1	0.3991 (2)	0.5025 (2)	0.34432 (14)	0.0044 (2)	
H1N	0.3634 (11)	0.5559 (12)	0.4165 (6)	0.0218 (13)	
H2N	0.3159 (12)	0.6073 (10)	0.2963 (7)	0.0212 (12)	
H3N	0.3294 (11)	0.3719 (9)	0.3435 (6)	0.0199 (12)	
C1	0.7711 (3)	0.3465 (3)	0.39746 (19)	0.0029 (3)	
C2	0.6484 (3)	0.4781 (3)	0.3163 (2)	0.0041 (3)	
H2A	0.7111 (11)	0.6233 (9)	0.3086 (7)	0.0220 (13)	
H2B	0.6871 (11)	0.4134 (12)	0.2420 (6)	0.0222 (13)	
Zn2	0.5000	0.5000	0.0000	0.0008 (3)	
O9	0.2958 (4)	0.3897 (3)	0.1320 (2)	0.0045 (4)	
H9A	0.2434 (11)	0.2593 (8)	0.1279 (6)	0.0175 (11)	
H9B	0.1606 (10)	0.4824 (9)	0.1443 (6)	0.0190 (11)	
O10	0.7911 (4)	0.3443 (4)	0.0509 (2)	0.0048 (4)	
H10A	0.7828 (12)	0.2322 (9)	0.1030 (6)	0.0180 (10)	
H10B	0.8989 (11)	0.2959 (10)	−0.0061 (6)	0.0197 (11)	
O11	0.5434 (4)	0.7298 (4)	0.0881 (2)	0.0054 (4)	
H11A	0.6938 (9)	0.7484 (9)	0.1036 (6)	0.0182 (11)	
H11B	0.4422 (10)	0.8523 (8)	0.0915 (7)	0.0200 (12)	

### Atomic displacement parameters (Å^2^)

**Table:**

	*U*^11^	*U*^22^	*U*^33^	*U*^12^	*U*^13^	*U*^23^
S1	0.0006 (10)	0.0009 (11)	0.0010 (18)	0.0001 (8)	−0.0003 (9)	0.0000 (11)
O1	0.0027 (6)	0.0037 (7)	0.0038 (10)	−0.0006 (5)	0.0016 (5)	0.0001 (7)
O2	0.0029 (6)	0.0028 (6)	0.0058 (11)	−0.0007 (5)	−0.0007 (6)	−0.0012 (7)
O3	0.0017 (6)	0.0040 (7)	0.0053 (11)	0.0018 (5)	0.0010 (6)	0.0000 (7)
O4	0.0039 (6)	0.0054 (7)	0.0018 (10)	0.0001 (5)	−0.0015 (6)	0.0011 (7)
Zn1	0.0015 (4)	0.0017 (4)	0.0017 (5)	−0.0001 (3)	−0.0001 (3)	0.0000 (3)
O5	0.0036 (7)	0.0071 (8)	0.0032 (11)	0.0014 (5)	−0.0002 (6)	0.0011 (7)
H5A	0.015 (2)	0.028 (3)	0.014 (3)	−0.0027 (18)	0.0046 (18)	0.002 (2)
H5B	0.015 (2)	0.019 (2)	0.016 (3)	0.0017 (15)	−0.0065 (17)	0.004 (2)
O6	0.0041 (7)	0.0064 (7)	0.0021 (11)	−0.0005 (5)	−0.0004 (6)	0.0002 (7)
H6A	0.0142 (18)	0.021 (2)	0.013 (3)	−0.0020 (15)	0.0034 (16)	−0.005 (2)
H6B	0.017 (2)	0.023 (2)	0.017 (3)	−0.0076 (17)	−0.0052 (18)	−0.005 (2)
O7	0.0042 (6)	0.0041 (7)	0.0026 (10)	−0.0011 (5)	−0.0004 (6)	0.0014 (7)
O8	0.0028 (7)	0.0091 (9)	0.0050 (12)	0.0008 (5)	0.0001 (6)	0.0022 (8)
N1	0.0030 (4)	0.0057 (5)	0.0041 (7)	0.0012 (3)	−0.0013 (4)	0.0004 (5)
H1N	0.017 (2)	0.033 (3)	0.015 (3)	0.002 (2)	0.0004 (19)	−0.009 (3)
H2N	0.019 (2)	0.020 (2)	0.025 (4)	0.0015 (18)	−0.011 (2)	0.006 (2)
H3N	0.018 (2)	0.015 (2)	0.027 (4)	−0.0066 (16)	−0.001 (2)	0.001 (2)
C1	0.0020 (5)	0.0034 (6)	0.0032 (9)	0.0000 (4)	−0.0011 (5)	0.0007 (6)
C2	0.0042 (6)	0.0047 (6)	0.0030 (9)	−0.0002 (4)	−0.0004 (5)	0.0017 (6)
H2A	0.020 (2)	0.013 (2)	0.033 (4)	−0.0078 (17)	−0.005 (2)	0.007 (2)
H2B	0.018 (2)	0.035 (3)	0.013 (3)	0.007 (2)	−0.0031 (19)	−0.006 (3)
Zn2	0.0008 (4)	0.0008 (4)	0.0009 (5)	−0.0001 (3)	−0.0001 (3)	0.0001 (3)
O9	0.0045 (7)	0.0035 (7)	0.0050 (11)	−0.0005 (5)	0.0005 (6)	0.0004 (7)
H9A	0.021 (2)	0.0115 (17)	0.021 (3)	−0.0064 (15)	−0.001 (2)	−0.001 (2)
H9B	0.0135 (19)	0.016 (2)	0.024 (4)	0.0064 (14)	0.0009 (18)	−0.001 (2)
O10	0.0039 (7)	0.0047 (7)	0.0050 (11)	0.0019 (5)	−0.0012 (6)	0.0010 (7)
H10A	0.024 (3)	0.015 (2)	0.013 (3)	0.0011 (17)	0.0002 (19)	0.0054 (19)
H10B	0.020 (2)	0.020 (2)	0.016 (3)	0.0065 (17)	0.0040 (19)	−0.002 (2)
O11	0.0033 (6)	0.0048 (7)	0.0086 (12)	0.0002 (5)	−0.0015 (6)	−0.0033 (8)
H11A	0.0091 (16)	0.022 (2)	0.025 (3)	−0.0030 (15)	−0.0057 (17)	−0.004 (2)
H11B	0.017 (2)	0.0118 (18)	0.031 (4)	0.0059 (14)	−0.003 (2)	−0.006 (2)

### Geometric parameters (Å, º)

**Table:**

S1—O3	1.473 (4)	N1—H1N	1.033 (7)
S1—O1	1.474 (5)	N1—C2	1.481 (2)
S1—O4	1.480 (6)	C1—C2	1.523 (4)
S1—O2	1.484 (4)	C2—H2A	1.085 (6)
Zn1—O5	2.039 (2)	C2—H2B	1.091 (7)
Zn1—O5^i^	2.039 (2)	Zn2—O10	2.067 (3)
Zn1—O6	2.093 (3)	Zn2—O10^ii^	2.067 (3)
Zn1—O6^i^	2.094 (3)	Zn2—O11^ii^	2.075 (2)
Zn1—O7^i^	2.173 (2)	Zn2—O11	2.075 (2)
Zn1—O7	2.173 (2)	Zn2—O9	2.129 (3)
O5—H5A	0.973 (7)	Zn2—O9^ii^	2.129 (3)
O5—H5B	0.997 (7)	O9—H9B	0.966 (6)
O6—H6A	0.981 (6)	O9—H9A	0.979 (5)
O6—H6B	0.985 (6)	O10—H10A	0.977 (8)
O7—C1	1.272 (4)	O10—H10B	0.979 (6)
O8—C1	1.240 (3)	O11—H11B	0.966 (6)
N1—H3N	1.022 (6)	O11—H11A	0.966 (6)
N1—H2N	1.027 (8)		
			
O3—S1—O1	110.2 (3)	O8—C1—O7	126.0 (3)
O3—S1—O4	110.1 (3)	O8—C1—C2	116.2 (2)
O1—S1—O4	109.5 (3)	O7—C1—C2	117.7 (2)
O3—S1—O2	109.3 (3)	H2A—C2—H2B	107.9 (7)
O1—S1—O2	109.3 (3)	H2A—C2—N1	109.5 (4)
O4—S1—O2	108.4 (3)	H2B—C2—N1	109.5 (4)
O5—Zn1—O5^i^	180.0	H2A—C2—C1	108.5 (5)
O5—Zn1—O6	88.59 (11)	H2B—C2—C1	109.7 (4)
O5^i^—Zn1—O6	91.41 (11)	O10—Zn2—O10^ii^	180.0
O5—Zn1—O6^i^	91.41 (11)	O10—Zn2—O11^ii^	91.57 (10)
O5^i^—Zn1—O6^i^	88.59 (11)	O10^ii^—Zn2—O11^ii^	88.43 (10)
O6—Zn1—O6^i^	180.0	O10—Zn2—O11	88.43 (10)
O5—Zn1—O7^i^	92.01 (9)	O10^ii^—Zn2—O11	91.57 (10)
O5^i^—Zn1—O7^i^	87.99 (9)	O11^ii^—Zn2—O11	180.0
O6—Zn1—O7^i^	93.38 (10)	O10—Zn2—O9	91.52 (11)
O6^i^—Zn1—O7^i^	86.62 (10)	O10^ii^—Zn2—O9	88.48 (11)
O5—Zn1—O7	87.99 (9)	O11^ii^—Zn2—O9	94.16 (10)
O5^i^—Zn1—O7	92.01 (9)	O11—Zn2—O9	85.84 (10)
O6—Zn1—O7	86.62 (10)	O10—Zn2—O9^ii^	88.48 (11)
O6^i^—Zn1—O7	93.38 (10)	O10^ii^—Zn2—O9^ii^	91.52 (11)
O7^i^—Zn1—O7	180.0	O11^ii^—Zn2—O9^ii^	85.84 (10)
H5A—O5—H5B	106.7 (6)	O11—Zn2—O9^ii^	94.16 (10)
H6A—O6—H6B	108.1 (6)	O9—Zn2—O9^ii^	180.00 (12)
C1—O7—Zn1	128.26 (18)	H9B—O9—H9A	106.2 (6)
H3N—N1—H2N	107.6 (6)	H10A—O10—H10B	106.1 (6)
H3N—N1—H1N	109.8 (7)	H11B—O11—H11A	111.4 (6)
H2N—N1—H1N	105.0 (6)		

Symmetry codes: (i) −*x*+1, −*y*, −*z*+1; (ii) −*x*+1, −*y*+1, −*z*.

### Hydrogen-bond geometry (Å, º)

**Table:**

*D*—H···*A*	*D*—H	H···*A*	*D*···*A*	*D*—H···*A*
O5—H5*A*···O4^iii^	0.973 (7)	1.793 (7)	2.755 (4)	169.0 (7)
O5—H5*B*···O8^iv^	0.997 (7)	1.656 (8)	2.642 (4)	168.9 (6)
O6—H6*A*···O3^v^	0.981 (6)	1.722 (6)	2.696 (3)	170.8 (5)
O6—H6*B*···O4^vi^	0.985 (6)	1.751 (5)	2.729 (3)	171.8 (7)
O9—H9*A*···O1^vi^	0.979 (5)	1.732 (5)	2.707 (3)	173.8 (6)
O9—H9*B*···O2	0.966 (6)	1.895 (6)	2.811 (3)	157.2 (6)
O10—H10*A*···O3^v^	0.977 (8)	1.740 (8)	2.713 (4)	173.0 (7)
O10—H10*B*···O2^ii^	0.979 (6)	1.811 (7)	2.745 (4)	158.5 (7)
O11—H11*A*···O2^vii^	0.966 (6)	1.772 (6)	2.726 (3)	168.6 (6)
O11—H11*B*···O1	0.966 (6)	1.824 (6)	2.750 (3)	159.5 (7)
C2—H2*A*···O2^vii^	1.085 (6)	2.682 (9)	3.351 (4)	119.4 (6)
C2—H2*A*···O5^viii^	1.085 (6)	2.716 (8)	3.489 (3)	127.9 (6)
C2—H2*B*···O10	1.091 (7)	2.579 (8)	3.649 (4)	166.7 (7)
N1—H1*N*···O7^viii^	1.033 (7)	1.853 (7)	2.848 (3)	160.8 (7)
N1—H2*N*···O4	1.027 (8)	1.961 (7)	2.877 (3)	147.0 (7)
N1—H3*N*···O6	1.022 (6)	2.216 (7)	3.066 (3)	139.5 (5)

Symmetry codes: (ii) −*x*+1, −*y*+1, −*z*; (iii) −*x*, −*y*+1, −*z*+1; (iv) *x*−1, *y*, *z*; (v) *x*+1, *y*−1, *z*; (vi) *x*, *y*−1, *z*; (vii) *x*+1, *y*, *z*; (viii) −*x*+1, −*y*+1, −*z*+1.
